# Castration the Remedy for Crime*Read before the Colorado State Medical Society.

**Published:** 1895-09

**Authors:** B. A. Arbogast

**Affiliations:** Breckenridge, Colo.


### Castration the Remedy for Crime. *

BY B. A. ARBOGAST, M. D., BRECKENRIDGE, COLO.

It may be I should ask not only your indulgence, but your pardon for the few remarks I have to make on the above subject.

The problem of how to care for our pauper and criminal classes is crowding itself upon all modern thinkers in all parts of the civilized world, and as population and wealth increase the pauper and criminal classes present to the sociologist, economist, and philanthropist a full realization of the fact that the only worthy aim of a system of relief is the restoration to the ranks of normal manhood and womanhood of those criminals who are capable of such restoration, and the speedy extinction of those who are beyond the possibility of such help; and again, we are well aware of the prudish sentiment of our people in speaking so radically of how to solve the problem. Crime becomes more revolting as civilization becomes more refined, despite the fact of constantly increasing facilities for the amelioration of the vicious and criminal classes in all communities; for never has there beeu a time in the history of the world, when each and every individual has had such an opportunity for self-improvement as now. We have the example of good homes, good schools, churches, reformatories, hospitals, jails, penitentiaries and the gallows finally, after the human brute had spread sorrow and desolation (often worse than death) to countless and untold innocent and pure and helpless of our society.

Vice and crime are nothing else than the sickness of the social body, and the physician is the true one to suggest a remedy and apply the treatment. Castration, with our antiseptic surgery and anesthesia is painless and safe, and would have a greater deterrent effect on the vicious than our penitentiaries or the gallows, besides leaving the subject as useful in many respects as before, minus his vicious impulses and a certainty that his kind would eventually be exterminated. I doubt if any one would object to the treatment being applied to the psycho-sexual monster, the Sadist; and the rapist has such a perverted sexual intinct that very often when once his lust is aroused, it is entirely beyond his control, and the greatest cruelty is resorted to, to accomplish his hellish design.

---

* Read before the Colorado State Medical Society.

Together we aim at the conservation of American liberties, a higher standard of American citizenship, the ends of universal justice, the evolution of a nobler humanity.

Government we must have, for defining and protecting civil rights; and it has an inherent right to determine what shall be the character and the rights of its citizens. Consequently, it has the right and also the obligation, to provide appropriate means and facilities for the formation of such a character for every citizen as the nature of the government requires.

We assume, therefore, that the proper treatment for all our citizens is such that will best fit the whole being, his physical first, so that his intellectual and moral being can be trained for the real obligations and duties of a citizen.

We have no right to assume, however, that because the individual has been created with all his animal desires in excess of his moral responsibility, that government shall not interfere by taking away his ability to perpetuate his kind and thereby reduce to the minimum the evil of the individual, and increase the security and comfort of the law-abiding citizens. For no punishment is complete which does not primarily aim to annihilate the evil with the least suffering to the individual. If national and state governments have a right to say what kind of quarantine to establish to save their people from some scourge or pestilence, or say what kind of sewage a city shall have to prevent disease, it is logical to say it has the right, and it is an obligation it owes to the upright and just to speedily exterminate certain classes of criminals.

It is not necessary to state to this Association the established fact of hereditary taint. It also seems to be a fact that among all the lower animals the plan outlined is carried to a successful issue, with happy results—while the divine human animal is entirely an accident, left to the caprice of chance—and when they have spread death and desolation all around them, they are compelled to commit suicide in Canon City, with only half the good accomplished that might be, had he been healed of his evil desires, and thereby having a very much greater influence over others. I repeat—the physician should, of all others, be the one to suggest the reform indicated, as every thought in the mind of a real physician, who is a true economist, should be directed toward the physical improvement of progeny. Witty criticisms and faithless conservatism must give way to truth and to intelligent opinion.

The human species in the highest type of animal life, and the highest development of man that has ever been attained was among the Greeks. Among the Spartans the greatest care was taken that there should be no propagation of those who were mentally or physically defective. The time has come in our civilization when there should be a revision of our laws to such an extent that it shall not only be unlawful for near of kin to marry, but that confirmed criminals and vagrants should be rendered incapable of reproducting their kind. It has only been a few days since that a notorious female thief, named Sophie Lyons, was said to be plying her vocation here in Denver, and her son was serving a term in an eastern State prison for crime, only to be released to be arraigned again for the same offense, and the posterity of such a woman is a constant menace to any city or State. All professional and confirmed criminals, before they are liberated, should undergo an operation that would effectively prevent their procreation. He was a wise man who said, "that to produce a high type of intellectual and physical development in the individual, it is necessary to begin two hundred years before the birth of a child." All breeders of domestic animals are extremely careful and particular in selecting their males and females for such purposes, and the inference to be drawn from this is that all men and women should not be allowed to mate or to marry. It is ours, as physicians, to teach the better doctrine and contemplate the loftiest and most inspiring spectacle of "federation of the world" on this point to which medicine is surely feeling its way.—*Denver Medical Journal*, *August*, *1895*.

